# Clinical Presentation, Pathogenesis, Diagnosis, and Treatment of Epidermolysis Bullosa Acquisita

**DOI:** 10.1155/2013/812029

**Published:** 2013-07-15

**Authors:** Ralf J. Ludwig

**Affiliations:** Department of Dermatology, University of Lübeck, Ratzeburger Allee 160, 23538 Lübeck, Germany

## Abstract

Epidermolysis bullosa acquisita (EBA) is a chronic mucocutaneous autoimmune skin blistering disease. The pathogenic relevance of autoantibodies targeting type VII collagen (COL7) has been well-documented. Therefore, EBA is a prototypical autoimmune disease with a well-characterized pathogenic relevance of autoantibody binding to the target antigen. EBA is a rare disease with an incidence of 0.2 new cases per million and per year. The current treatment of EBA relies on general immunosuppressive therapy, which does not lead to remission in all cases. Therefore, there is a high, so far unmet medical need for the development of novel therapeutic options. During the last 10 years, several novel in vitro and in vivo models of EBA have been established. These models demonstrated a critical role of the genetic background, T cells, and cytokines for mediating the loss of tolerance towards COL7. Neutrophils, complement activation, Fc gamma receptor engagement, cytokines, several molecules involved in cell signaling, release of reactive oxygen species, and matrix metalloproteinases are crucial for autoantibody-induced tissue injury in EBA. Based on this growing understanding of the diseases' pathogenesis, several potential novel therapeutic targets have emerged. In this review, the clinical presentation, pathogenesis, diagnosis, and current treatment options for EBA are discussed in detail.

## 1. Clinical Presentation of Epidermolysis Bullosa Acquisita

In the beginning of the 20th century, the term “epidermolysis bullosa acquisita” (EBA) was used as a descriptive clinical diagnosis for patients with adult onset and features resembling those of hereditary dystrophic epidermolysis bullosa [1]. Almost 70 years later, EBA was distinguished from other bullous diseases on the basis of distinctive clinical and histological features, implementing the first diagnostic criteria for the disease. Specifically, these included (i) clinical lesions resembling epidermolysis bullosa dystrophica, (ii) adult onset of disease, (iii) a negative family history of epidermolysis bullosa dystrophica, and (iv) exclusion of other bullous diseases [2]. Based on the current understanding of EBA pathogenesis, additional/other criteria define EBA diagnosis today (see Section 13). The cutaneous manifestations in EBA patients are heterogeneous. However, EBA patients can be classified into two major clinical subtypes: noninflammatory (classical or mechanobullous) and inflammatory EBA, which is characterized by cutaneous inflammation resembling bullous pemphigoid, linear IgA disease, mucous membrane pemphigoid, or Brunsting-Perry pemphigoid [3–5]. The clinical presentation of an individual EBA patient may change during the course of the disease, or the same patients may present with two different forms simultaneously.

## 2. Mechanobullous EBA

The mechanobullous (noninflammatory, classical) variant of EBA is observed in approximately 1/3 of the patients (Table 1) and is characterized by skin fragility, tense blisters, scaring, and milia formation preferably localized to trauma-prone sites and the extensor skin surface. In these patients, nail dystrophy and postinflammatory hyper- and hypopigmentation are also frequently observed. Furthermore, the oral mucosa is often affected in these patients. In mild cases, the clinical presentation is similar to porphyria cutanea tarda, while severe cases are comparable to hereditary recessive dystrophic epidermolysis bullosa [6–9]. 

## 3. Inflammatory-Type EBA

The inflammatory variants of EBA, accounting for approximately 2/3 of the cases (Table 1), clinically mimic other autoimmune bullous dermatoses (AIBDs) such as bullous pemphigoid (BP), linear IgA disease (LAD), mucous membrane pemphigoid (MMP), or Brunsting-Perry pemphigoid. In patients with inflammatory EBA, widespread vesiculobullous eruptions are observed, typically involving the trunk, central body, extremities, and skin folds. Usually the patients suffer from pruritus [6–8].

## 4. Congenital EBA

During pregnancy, IgG is transferred from the mother to the fetus [10]. Therefore, the transfer of anti-COL7 IgG could potentially induce EBA in neonates of mothers with EBA. Interestingly, so far only one case of this congenital EBA has been reported: a mother diagnosed with EBA delivered an otherwise healthy girl, who was noted at birth to have tense blisters and areas of denuded skin. The patient's skin lesions progressed, and the dermatology service was consulted. Direct immunofluorescence of perilesional skin revealed linear deposition of IgG and C3, and ELISA detected anti-COL7 autoantibodies, confirming the diagnosis of EBA. Based on the assumption of disease induction by placental autoantibody transfer, supportive treatment was initiated. This led to cession of new blister formation within 10 days and healing of all erosions within 2 months [11].

## 5. Extracutaneous EBA Manifestations and Associated Diseases

### 5.1. Extracutaneous EBA Manifestations

Extracutaneous EBA manifestations include ocular, oral mucosa, esophagus, anal, vaginal, tracheal, and laryngeal lesions [3, 4, 12]. Ocular involvement in EBA predominantly presents with scaring, resembling lesions observed in patients with MMP. It may be the only EBA manifestation in a patient or may be observed in addition to other lesions. In severe cases, ocular involvement may lead to blindness. Interestingly, the predominant Ig class in ocular EBA seems to be IgA; that is, most cases reported noted IgA deposits at the basement membrane. Several EBA patients with ocular involvement have been described. However, the proportion of EBA patients affected by eye lesions has not been systemically evaluated [13–18]. In EBA, erosions and blistering can also affect the oral mucosa. Oral lesions are most commonly observed in patients with noninflammatory EBA and those with MMP-like or LAD-like inflammatory EBA [19, 20]. Esophageal strictures are another extracutaneous manifestation and severe complication in EBA patients. Patients may not be able to swallow foods and thus require endoscopic esophageal dilations, which may have to be repeated several times, if disease activity cannot be controlled [21–23]. Rarely, laryngeal involvement may cause hoarseness, impaired phonation, and loss of voice, and may lead to irreversible respiratory distress. These may be caused by trauma induced by intubation [24, 25]. 

The expression of COL7 in most of the anatomic regions affected by EBA other than the skin [26–29] may best explain occurrence of these extracutaneous EBA manifestations. Overall, the prevalence of mucosal lesions in EBA is largely unknown. However, one study evaluated the mucosal involvement in 4 EBA patients in a multidisciplinary approach that included dermatologists, ophthalmologists, radiologists, and otolaryngologists. All the 4 patients showed presence of multiple mucosal lesions. Specifically, all had oral lesions ranging between erosions, blisters, tooth loss, and mandibular contraction resulting in impaired ability to open the mouth and alveolar bone loss. Lesions in the nasal mucosa were also noted in all patients, ranging from dryness to extensive fibrosis and synechiae. Symblepharon was also noted in all the patients, as well as pharyngeal and laryngeal strictures. The esophagus dysmotility and/or strictures were observed in 3 of the 4 patients. One patient also suffered from perianal lesions, and another patient presented with erosions on the penile shaft. Hence, these findings have generated awareness to regularity screen EBA patients for (even subclinical) mucosal disease, as the delay of appropriate treatment may lead to severe complications as outlined previously [4, 25]. 

### 5.2. Diseases Associated with EBA

On a case report basis, EBA has been noted to be associated with diabetes mellitus cryoglobulinemia, subacute cutaneous/systemic lupus erythematosus, and psoriasis [3, 4, 30]. Interestingly, regarding the association of EBA with psoriasis, in all so far described 4 patients, EBA manifested after psoriasis [31–34]. However, most of these findings are anecdotal reports, and no clear pathogenic interaction of EBA with these diseases has been established. In contrast, accumulating evidence suggests that EBA and inflammatory bowel diseases (IBDs) such as ulcerative colitis (UC) and Crohn's disease (CD) are associated. More specifically, IBD has been reported to be present in approximately 30% of patients with EBA. However, it needs to be taken into account that many of these observations were made before modern diagnostic criteria for EBA were established [35–38]. So far, CD has been noted to be associated with EBA in at least 25 cases [39, 40] as well as four cases of EBA associated with UC [40]. Furthermore, in patients with active IBD, circulating antibodies to COL7 have been noted in frequencies ranging from 6 to 60% [41–43]. Unpublished data from our laboratory indicates that the incidence of anti-COL7 IgG in patients with IBD is at the lower end of the published incidences. Further evidence of a pathogenic link between IBD and EBA has been obtained in EBA mouse models: in both antibody transfer-induced EBA [44] and immunization-induced EBA [45, 46] in addition to skin blistering, blister formation was observed in esophagus, stomach, small intestine, and colon. The prevalence of blister formation in these mouse models paralleled the expression of COL7, which continuously decreased from the one observed in the oral cavity to the distal colon. This anti-COL7-induced gastrointestinal tissue injury is of functional relevance, as a reduced body weight in diseased mice was observed [28].

## 6. Epidemiology

**Figure 1:**
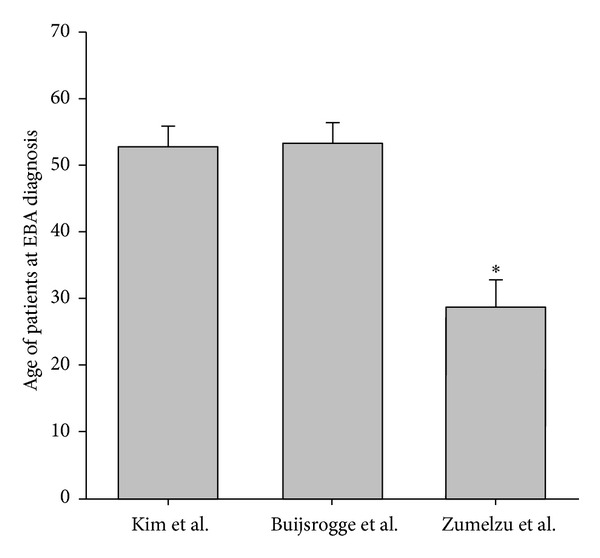
Mean age of EBA onset in Korea, Netherlands, and blacks with African descent. Mean (SEM) patient age (years) at the diagnosis of EBA in 3 large cohorts of patients from Korea, Netherlands, and France (blacks with African descent) [6–8]. ∗ indicates *P* < 0.05 (ANOVA).

EBA is a rare disease with an incidence ranging from 0.2 to 0.5 new cases per million and per year [47–49]. As EBA is a chronic disease, the prevalence is presumably higher, but data on EBA prevalence is not available. Three studies have reported epidemiological data from EBA patients [6–8]: this data from a total of 83 patients shows that EBA affects females slightly more often than males. Furthermore, median age of onset is 47 years (75 percentile: 30–66 years). Yet, EBA has also been diagnosed in children [8, 50, 51], and approximately 20% of EBA patients are over 70 years old. Interestingly, black EBA patients of African descent are significantly younger compared to EBA patients in Korea or the Netherlands (Figure 1). Regarding the different clinical phenotypes, the inflammatory variants are observed most commonly (67%), while classical EBA is observed in 33% of the patients. This epidemiological data on EBA is summarized in Table 1. 

## 7. Pathogenesis of EBA

Like observed in all other AIBD, in EBA, autoantibodies targeting antigens located within the skin mediate tissue injury [5, 52]. Since the discovery of COL7 as the autoantigen in EBA [53], the development of model systems duplicating several aspects of the human disease has greatly contributed to our current understanding of the diseases' pathogenesis. 

## 8. Identification of Type VII Collagen as the Autoantigen in EBA

**Figure 2:**
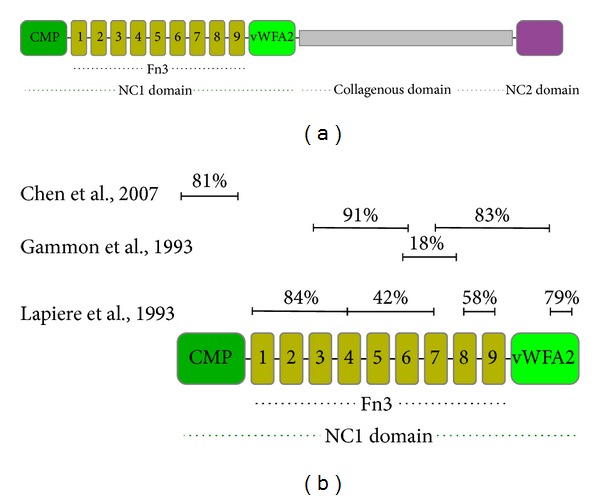
Fine mapping of the autoantibody reactivity in epidermolysis bullosa acquisita. (a) Schematic structure of COL7. In vivo, COL7 chains form a trimer. Single COL7 chain consists of one central collagenous domain, flanked by 2 noncollagenous (NC) domains. The N-terminal NC-1 domain consists of several subdomains with high homologies to adhesion proteins (CMP: cartilage matrix-like, Fn3: fibronectin 3-like, and vWFA2: von Willebrand factor A-like). The C-terminal NC2 domain contains conserved cysteine residues, which accompany an antiparallel assembly of these collagen molecules. The COL7 gene (COL7A1) maps to the locus 3p21 [70–72]. (b) Summary of epitope mapping studies performed within the NC1 domain. The percentages indicated in the figure correspond the percentage of patients with autoantibodies to their regions.

COL7 was identified as the autoantigen in EBA approximately 30 years ago. In 1984, Dr. Woodley and colleagues identified a 290 kDa protein located at the basement membrane of human skin, which was distinct from all other known components of the basement membrane [54]. Four years later, the same group identified the carboxyl terminus of COL7 as the autoantigen in EBA [53]. In most EBA patients, the autoantibodies are IgG. In addition to IgG, IgA anti-COL7 autoantibodies are observed either as the only Ig class or in combination with IgG autoantibodies (Table 1). Detailed epitope mapping studies with sera from EBA patients [55–57] demonstrated that most autoantibodies bind to epitopes located within the noncollagenous (NC) 1 domain of COL7 (Figure 2). In only very few patients, antibody reactivity to either the collagenous domain [58] or the NC2 domain [59] can be detected.

## 9. Demonstration of the Pathogenic Relevance of Anti-COL7 Antibodies

**Figure 3:**
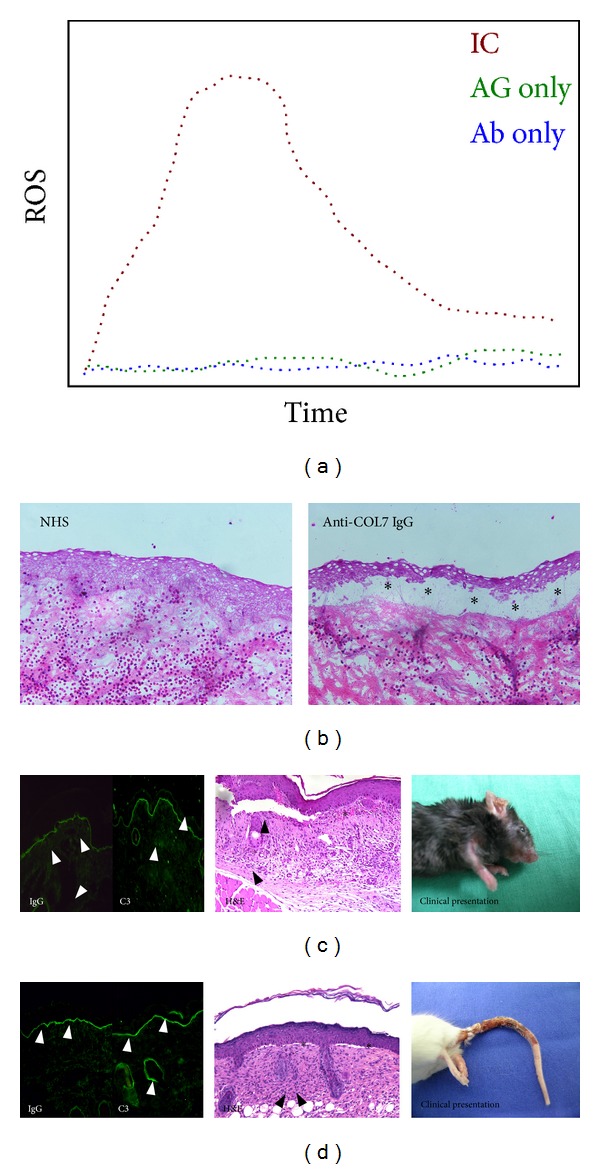
Demonstration of the pathogenicity of anti-type VII collagen antibodies. (a) Immune complexes (IC) of anti-COL7 IgG (Ab) and COL7 (AG) are able to activate neutrophils isolated from either healthy humans or mice. Endpoint measurement is the release of reactive oxygen species (ROS) by chemiluminescence. Detection of elastase by ELISA is an additional measurement for neutrophil activation in this assay. For AIBD, this assay was first described for type XVII collagen and anti-type XVII collagen antibodies obtained from patients with bullous pemphigoid [73]. Later, the same method was adopted for EBA [61]. The figure shows a schematic result (ROS release) from neutrophils activated with either antigen or antibody alone, or with immune complexes. (b) Incubation of cryosections of normal human or mouse skin with antibodies targeting COL7, followed by incubation with neutrophils from healthy donors induces dermal-epidermal separation. This so-called “cryosection assay” also allows to investigate neutrophil activation. This assay was initially developed using sera from BP patients [74, 75] and was later adopted for EBA [60]. Shown are H&E stained sections of cryosections of normal human skin incubated with either normal human serum (NHS) or IgG from an EBA patient. After the washing of antibodies, neutrophils from healthy human donors were added to the sections. While no pathology is observed in the section incubated with NHS, a clear separation of the epidermis from the underlying dermis is present in the section incubated with anti-COL7 IgG. ∗ indicates split formation. (c) Injection of rabbit or human anti-COL7 IgG into mice leads to IgG and complement (C3) deposition along the dermal-epidermal junction (arrow heads). Subsequently, subepidermal blistering (∗) accompanied with a dermal leukocyte infiltration (arrow heads) develops. Clinically, mice present with erythema, crusts, and alopecia. Fresh blisters are rarely observed, most likely due to the thin epidermis in mice. This antibody transfer (passive) EBA mouse model was first described independently in 2005 by two groups [44, 63]. The histology and images are from C57Bl/6 mice injected with rabbit anti-COL7 IgG, 12 days after the first antibody injection. (d) Immunization of susceptible mouse strains with recombinant proteins located within the NC1 domain leads to autoantibody production, which can be detected by direct IF microscopy from skin biopsies. Subsequent to autoantibody deposition at the dermal-epidermal junction, complement activation (evidenced by C3 deposition at the dermal-epidermal junction) and histological and clinical findings duplicating the human disease are observed.

The pathogenic relevance of anti-COL7 antibodies has been demonstrated in vitro, ex vivo, and in vivo. In detail, in vitro pathogenicity was demonstrated by the incubation of cryosections of human skin with serum, total IgG, or NC1-affinity purified IgG isolated from EBA patients. This led to a linear IgG deposition along the dermal-epidermal junction. Sole antibody binding did not however induce a separation of the epidermis from the dermis. When these sections were subsequently incubated with neutrophils from healthy blood donors, this led to the location of neutrophils to the dermal-epidermal junction, followed by the release of reactive oxygen species (ROS) from the neutrophils and ultimately a separation of the epidermis from the dermis (Table 2, Figure 3) [60]. In addition to the demonstration of the pathogenic relevance of autoantibodies directed against COL7, these experiments also documented the key contribution of neutrophils to blister induction. Based on this insight, neutrophils from healthy blood donors were isolated and incubated with fixed immune complexes of anti-COL7 IgG and recombinant COL7. This led to neutrophil activation, assayed by determination of ROS [61]. 

Subsequently, two groups independently documented the pathogenic relevance of autoantibodies targeting COL7 in 2005. Sitaru and colleagues [44] immunized rabbits with recombinant proteins located within the NC1 domain of COL7. Injection of the total immune IgG isolated from these rabbits into C57Bl/6 or BALB/c mice induced subepidermal skin blisters, reproducing human EBA at the clinical, histological, electron microscopical, and immunopathological levels (Table 2, Figure 3). Also in 2005, Woodley and colleagues generated rabbit anti-human COL7 IgG, which also induced experimental EBA when injected into outbred hairless SKH1 mice [63]. This blister-inducing activity of anti-COL7 IgG has been confirmed by similar experiments; that is, experimental EBA can be induced by the injection of (i) affinity-purified anti-COL7 antibodies from EBA patients' sera into adult hairless mice [64], (ii) affinity-purified anti-CMP EBA patient antibodies injected into hairless mice [57], (iii) rabbit anti-human COL7 IgG directed against the Fn3-like repeats of COL7 into hairless mice [65], (iv) rabbit anti-mouse vWFA2 IgG injected into several, but not all, strains of in- and outbred mice [66], and (v) injection of rabbit anti-human COL7 IgG into COL7-humanized mice [67, 76]. Most of these antibody-transfer models of EBA mimic the inflammatory variants of the disease. However, in some models, nail loss is additionally observed [63, 64], which is mostly observed in mechanobullous EBA. 

**Figure 4:**
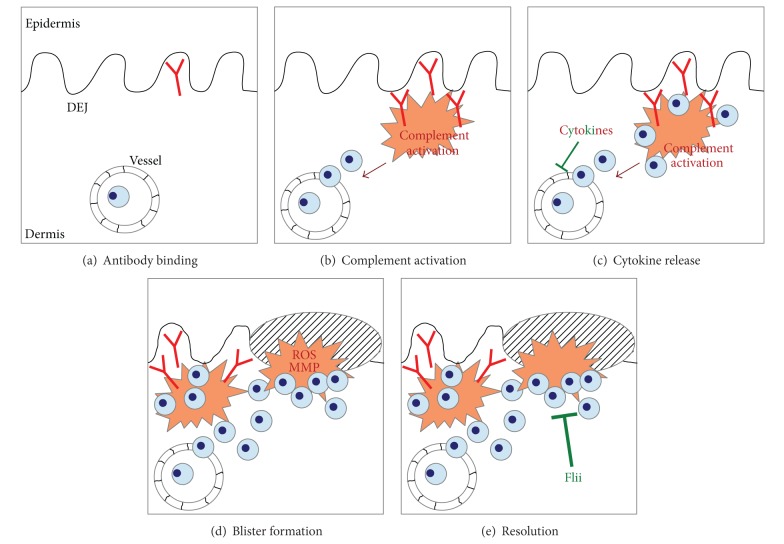
Pathogenesis of autoantibody-induced tissue injury in EBA. (a) In EBA, blister formation is induced by antibody binding to COL7 located at the dermal-epidermal separation. (b) This leads to the activation of the complement system. Cleavage products of complement activation, for example, C5a, mediate CD18-dependent neutrophil extravasation into the skin. (c) Subsequently, cytokines are released from yet to be defined cells. This can either contribute to tissue injury (GM-CSF, IL-1, CXCL1, and CXCL2) or have potent anti-inflammatory effects (IL-6, IL-1ra). (d) Blister formation is mediated by MMP such as elastase and reactive oxygen species (ROS) released from neutrophils after binding to the immune complexes. (e) Recent attention has focused on the resolution of inflammation in EBA. Flightless I (Flii) has potent anti-inflammatory and proresolving effects in experimental EBA.

In an attempt to model all aspects of the human disease, several mouse strains were immunized with a protein spanning the amino acid residues 757–967 of murine NC1, which contains a GST-tag for isolation, termed GST-mCOL7C (Figure 3). Mice were immunized with the protein and were subsequently boosted 3 times at 3-week intervals. This induced clinical disease in over 80% of SJL/J and approximately 50% in BALB/c and Fc gamma receptor IIB-deficient mice on a C57Bl/6 genetic background (Table 2, Figure 4). In contrast, SKH1 and C57Bl/6 mice did not develop skin blistering [45]. Experimental EBA can also be induced by immunization in (i) susceptible inbred mouse strains (SJL/J, B6.SJL-H2s, B10.s, and MRL/MpJ) by single immunization with GST-mCOL7C [68], (ii) an autoimmune-prone advanced intercross mouse line (AIL) by single immunization with GST-mCOL7C [77], and (iii) in SJL/J and B6.SJL-H2s mice by single immunization with another immunodominant protein corresponding the vWFA2-like subdomain of COL7s' NC1 domain [66].

## 10. Mechanisms Leading to Loss of Tolerance to COL7

Due to the previous lack of appropriate models [78, 79], investigations on the mechanisms leading to the loss of tolerance have just begun. Clinical observations and MHC genotyping in EBA patients point towards a genetic control of EBA, which was also documented in animal models of the disease. Regarding cellular requirements for the induction of autoantibody production, CD4 T cells have been identified to be crucial. Furthermore, in experimental EBA, the proliferation of CD4 T cells depends on heat-shock protein (HSP) 90. These findings are presented and discussed in detail within the following paragraphs (Table 3). 

### 10.1. EBA Susceptibility Is Genetically Controlled

EBA susceptibility is linked to certain MHC alleles in patients. One study documented an association with HLA-DR2 [80], and in a later study, HLA-DRB1*15:03 was found to be associated with EBA [8]. The observation of cooccurrence of EBA cases in one family further points towards a genetic control of EBA [81]. An MHC restriction is also observed in immunization-induced experimental EBA in mice: after a single immunization with GST-mCOL7C, most inbred mouse strains developed anti-COL7 antibodies. However, only few strains develop subsequently develop clinically manifest skin blistering. Among those, SJL/J and B6.SJL-H2s mice show a high incidence of severe disease, and C57Bl/10.s and MRL/MpJ mice develop mild EBA at a low incidence. NOD/Shilt/J and C57Bl10.q mice were resistant to both anti-COL7 autoantibody production and clinical disease development [68]. 

Recently, in a cohort of EBA patients diagnosed in a French referral center for autoimmune bullous dermatoses (AIBDs), 54% were black skinned, while only 3% of other AIBD patients were black skinned. Therefore, black-skinned patients have a significantly higher risk to develop EBA, pointing towards a genetic predisposition outside the MHC locus [8]. This assumption is supported by several observations in immunization-induced EBA: in this model, SJL/J, B6.SJL-H2s, and C57Bl/10.s mice are susceptible to disease induction. All strains share the H2s locus but differ genetically regarding genes outside this locus. After immunization, EBA incidence and clinical disease severity are different among these strains [68]. Furthermore, immunization-induced EBA-resistant C57Bl/6 mice become susceptible if they lack the expression of the inhibitory Fc gamma IIB receptor [44]. Overall, these differences indicate that genes outside the MHC locus contribute to EBA susceptibility. In order to define this genetic control of EBA susceptibility, an advanced autoimmune prone intercross mouse line (AIL) was generated. Immunization-induced EBA-resistant parental mouse stains (NZM2410/J, BXD2/TyJ, and Cast) and MRL/MpJ EBA-susceptible mice were intercrossed at an equal strain and sex distribution [82]. Mice of the 4th offspring generation (G4) were then immunized with GST-mCOL7C. Of these genetically diverse mice, 33% developed clinically manifest experimental EBA, while the remaining mice remained clinically healthy. These mice were then genotyped for 1400 single-nucleotide polymorphisms (SNPs) to identify genomic regions controlling EBA susceptibility (quantitative trait locus (QTL)). This led to the identification of several non-MHC QTL controlling EBA susceptibility [77]. Overall, clinical observations and data from experimental models clearly document a contribution of genes in- and outside the MHC locus to EBA susceptibility (Table 3).

### 10.2. CD4 T Cells Are Required to Induce Autoantibody Production

T-cell-deficient SJL^nude^ mice are completely protected from induction of immunization-induced EBA. In these mice, disease susceptibility could be restored by the transfer of T cells from wild type SJL/J mice that had been immunized with COL7 [83]. Hence, T cells are required for the induction of autoantibody production and possibly also for the maintenance of autoantibody production, as described for other AIBDs such as bullous pemphigoid and pemphigus vulgaris [85–89]. In order to further define T-cell subsets involved in the generation of anti-COL7 antibodies in experimental EBA, CD4- and CD8 T cells were depleted in a time restricted fashion. Depletion of CD4 T cells for two weeks starting one day prior to immunization significantly delayed both autoantibody production and the onset of clinical disease. In contrast, depletion of CD8 T cells for the same time period had no effect [66]. Therefore, CD4 T cells are required for the induction of autoantibody production in experimental EBA (Table 3). It is currently unknown which antigen-presenting cells (APCs) lead to the clonal selection and expansion of COL7 reactive CD4 T cells. 

### 10.3. HSP90 Modulates Autoantibody Production

Challenging the hypothesis that heat-shock protein 90 (Hsp90) inhibition induces cell death of autoreactive plasma cells (as described for malignant plasma cells [90]), mice were treated with HSP90 inhibitors before the induction of immunization-induced EBA (Table 3). In a therapeutic setting, mice with already established immunization-induced EBA were also treated with HSP90 inhibitors. Both HSP90 inhibitors prevented the onset of immunization-induced EBA and also ameliorated clinical disease in already established EBA. Furthermore, HSP90 inhibition led to the suppression of autoantibody production and reduced the dermal leukocyte infiltration. Unexpectedly, total plasma cell numbers, COL7-specific plasma cells, and germinal center B cells were unaffected by HSP90 blockade. Interestingly, T-cell proliferation was significantly inhibited, as evidenced by the reduced response of isolated lymph node cells from immunized mice to in vitro restimulation with anti-CD3/CD28 antibody or autoantigen in the presence of HSP90 inhibitors. These results indicated that HSP90 inhibition has no impact on normal or autoreactive plasma cells in vivo and identified T cells as targets of HSP90 blockade in EBA [84].

## 11. Autoantibody Production and Maintenance of a High Half-Life of Autoantibodies

### 11.1. Generation of Anti-COL7 IgG Is Restricted to Peripheral Lymph Nodes in Experimental EBA

In immunization-induced EBA, antigen-specific T cells can be detected in most secondary lymphoid organs (unpublished observation). Interestingly, antigen-specific B cells can only be detected in peripheral lymph nodes [84]. Therefore, COL7 autoantibodies are exclusively produced at this anatomical location. Regarding human EBA patients, the location of autoreactive B cells is unknown. In other AIBD, such as pemphigus vulgaris, antigen-specific B cells can be isolated from blood [91]. Therefore, it is tempting to speculate that anti-COL7 producing B cells are also found in the circulation of EBA patients. This assumption is supported by the detection of COL7-specific T cells in the circulation of EBA patients [92].

### 11.2. Production of Disease-Inducing Anti-COL7 IgG Is Associated with a Th1 Polarization in Peripheral Lymph Nodes in Experimental EBA

In immunization-induced EBA, most strains develop circulating and tissue-bound anti-COL7 antibodies. However, only few strains develop an overt blistering disease. Comparing the autoantibody response of clinically healthy versus diseased mice after COL7 immunization showed that IgG2 antibodies are associated with clinical EBA manifestation [45, 68, 69]. This antibody response reflects Th1 polarization of the immune response. This assumption is further supported by an increased IFN-*γ*/IL-4 ratio in the draining lymph nodes of EBA-susceptible mice, compared with EBA-resistant strains [69]. 

### 11.3. The Neonatal Fc Receptor Controls the Half-Life of Anti-COL7 Autoantibodies

After the autoantibodies are released into the circulation, their half-life is controlled by the neonatal Fc receptor (FcRn). The FcRn is an MHC class I-like molecule that, among other functions, protects IgG from catabolism [10]. In line, blockade of the FcRn leads to an enhanced clearance of all IgG, including autoantibodies. In animal models of AIBD, including pemphigus, bullous pemphigoid, and EBA, mice are completely blocked from disease induction [93, 94]. This protection can however be overridden by the transfer of excess amounts of antibodies [94]. 

## 12. Mechanisms of Autoantibody-Induced Tissue Injury

Experiments employing several model systems have identified several cellular and molecular requirements for autoantibody-induced tissue injury in EBA. This, for example, includes neutrophils, Fc receptors, complement activation, and cytokines. While the contribution of each of these cells and molecules has been well documented, the exact timely and spatial sequence of events leading to blister formation has not been determined in detail. Based on the current understanding of EBA pathogenesis, autoantibody-induced tissue injury in EBA can be divided into the following events: (i) binding of the autoantibodies to their target antigen, (ii) Fab- and Fc-dependent effects on blister formation, including the release of several cytokines, (iii) integrin-dependent extravasation of neutrophils into the skin, (iv) neutrophil activation, including the release of ROS and MMP, and (v) resolving of the autoantibody-induced tissue injury. 

### 12.1. Autoantibody-Induced Tissue Injury Is Genetically Controlled

In other animal models of antibody-induced inflammatory disease, for example, the K/BxN transfer arthritis model, disease susceptibility differs in inbred mouse strains [95]. In line, large differences in clinical disease severity were observed after the transfer of anti-COL7 IgG into different mouse strains. In C57Bl/6 mice, close to 30% of the body surface area was affected by EBA skin lesions 20 days after the first administration of anti-COL7 IgG. In contrast, a lower clinical disease activity was observed in BALB/c mice, while MRL/MpJ and outbred SKH1 mice were completely resistant to EBA induction by the transfer of anti-COL7 IgG. Overall, this points towards a (yet to be determined) genetic control of autoantibody-induced tissue injury in experimental EBA. This assumption is further supported by the observation of a high variation of skin blistering in outbred AIL mice after the transfer of anti-COL7 IgG [66]. 

### 12.2. Anti-COL7 Antibodies Rapidly Bind to Their Target Antigen

Morphological investigations on anti-COL7 IgG deposition after transfer into mice by direct immunofluorescence (IF) microscopy showed linear IgG deposits along the dermal-epidermal junction within 24 hours [28]. To further define the kinetics of anti-COL7 IgG deposition to the skin, autoantibodies were fluorescently labeled. These labeled anti-COL7 autoantibodies fully retained their ability to induce blistering in mice. These fluorescently labeled anti-COL7 autoantibodies were i.v. injected into mice, and their binding to the skin was observed using multiphoton in vivo microscopy using an established experimental setup [96]. Within minutes after i.v. injection, anti-COL7 IgG bound to the dermal-epidermal junction (*Iwata et al., unpublished*). To study clearance rates of circulating and tissue-bound autoantibodies to COL7 in experimental EBA, retention times of diaplacentally transmitted anti-COL7 autoantibodies in serum and in skin were investigated. Immediately after birth, comparable levels of pathogenic antibody concentrations were observed in maternal and neonatal mice. The clearance time of skin-bound autoantibodies was twice as long (8 weeks) as that of circulating autoantibodies (weeks). Interestingly, despite IgG and complement (C3) deposition along the dermal-epidermal junction in the neonatic mice, neither histological, or clinical alterations were observed [46]. In addition to the skin, immunoperoxidase and immunofluorescence staining analyses found COL7 expression to be confined to the basement membranes beneath other stratified squamous epithelia; for example, oesophagus, buccal, anal, and vaginal mucosae, but not in colonic mucosa [26, 27]. On the contrary, others noted scattered COL7 staining in colonic epithelium and in the small intestinal epithelial basement membrane [97, 98]. Furthermore, anchoring fibrils were detected by electron microscopy in the intestine [97], and COL7 expression was detected in oesophagus and colon [41, 99]. In mice, COL7 mRNA and protein can be detected in the skin and in all organs of the gastrointestinal tract. Of note, COL7 expression decreases from proximal to distal parts of the gastrointestinal tract [3].

### 12.3. F(ab)-Mediated Effects on Blister Formation

As mentioned previously, IgG antibodies from EBA patients bind to a large variety of epitopes located with the NC1 domain of COL7 (Figure 3). F(ab)-mediated effects on blister formation have been described in bullous pemphigoid, for example, a weakening of cell attachment after the binding of anti-BP180 antibodies to keratinocytes [100–102]. In contrast, no data on F(ab)-mediated effects of anti-COL7 antibodies on keratinocyte adhesiveness has been reported. As the NC1 domains multiple binding sites to other proteins located within the dermal-epidermal junction, it seems plausible that autoantibody binding interferes with these interactions [70, 103]. This may explain the pathogenesis of noninflammatory, mechanobullous EBA; however, this assumption needs to be experimentally confirmed.

### 12.4. Fc-Mediated Effects on Blister Formation

Fc-mediated effects after the binding of anti-COL7 to its autoantigen have been investigated in detail. Several lines of evidence suggest that the Fc portion of anti-COL7 antibodies is a key molecular prerequisite to initiate blister formation. In vitro, only IgG, but not F(ab)2 fragments, directed to COL7 induced dermal-epidermal separation when incubated on cryosections of human skin, followed by the addition of neutrophils from healthy donors [60]. Furthermore, not all isoclasses of anti-COL7 have the potential to induce dermal-epidermal separation. Specifically, only IgG1 and IgG3, but not IgG2 and IgG4, induced ex vivo blister formation [62]. Similar observations are made in mice. While injection of rabbit anti-COL7 IgG induces skin blistering, the administration of corresponding F(ab)2 fragments causes no pathology, despite binding to the dermal-epidermal junction [44]. The absence of skin lesions in mice injected with chicken anti-mouse COL7 IgY, which does not bind murine complement and Fc receptors, further underlines the importance of Fc-Fc receptor interactions for blister formation in experimental EBA [104]. Recent attention has also been attributed to the glycosylation status of IgG, as this has been demonstrated to have a great impact on IgG function [105]. Alterations in the Fc glycosylation patterns have been demonstrated in several chronic inflammatory diseases [106], and modification of IgG glycosylation has been demonstrated to have preventive and/or therapeutic effects in models of inflammation, including EBA [107–114].

The exact timely and special resolution of events occurring after autoantibody binding in EBA has yet to be defined. The current concept of antibody-induced tissue injury is that of a multistep, directed, but not linear, process leading from antibody binding to blister formation. So far, the following cells and molecules have been identified to contribute to blister formation in experimental EBA: (i) cytokines including IL-1, IL-6, GM-CSF, CXCL1, and CXCL2, (ii) complement activation, (iii) adhesion molecules, such as beta-2 integrins, (iv) Fc gamma receptors, (v) neutrophil activation, and (vi) resolving of the inflammatory response. These pathways are discussed later (Figure 4, Table 4). 

### 12.5. Differential Effects of Cytokines to Autoantibody-Induced Tissue Injury

There is an ample evidence for an increased expression of several cytokines in AIBD [115]. Despite these findings, and in contrast to other chronic inflammatory diseases [116–120], a cytokine-targeting therapy has not been established in EBA or any other AIBD—with the exception of the relatively well-documented beneficial effects of TNF*α* inhibition in mucous membrane pemphigoid [121, 122]. Regarding EBA, serum and skin IL-6 expression are increased. Compared to control sera, other cytokines were also increased in EBA but, most likely due to a high degree of variation, were not significantly different [123]. In experimental EBA, induced by the transfer of anti-COL7 IgG into C57Bl/6 or BALB/c mice, increased serum concentrations of several cytokines were noted. Specifically, elevated serum concentrations of TNF*α*, MIP-1*α*, GM-CSF, IL-1*α*, IL-1*β*, IL-4, IL-6, IL-10, IL-17, RANTES, and KC, but not MIP-1*β*, G-CSF, IL-2, IL-3, IL-5, IL-9, IL-12, IL-13, eotaxin, IFN-*γ*, and MCP-1, were observed in experimental EBA (Table 5) [123]. Furthermore, gene expression profiling of skin obtained from mice with experimental EBA additionally identified an increased cutaneous expression of IL-24, CXCL3 (GRO3), and CXCL5 (ENA-78) [124].

Functional studies documented little to no contribution of TNF*α* and MIP-1*α* in autoantibody-induced tissue injury in experimental EBA (*unpublished data*). As neutrophils are required to induce skin blistering in mice [126], the contribution of cytokines affecting neutrophil functions such as IL-8 (CXCL1 and CXCL2 in the mouse) and GM-CSF were next evaluated for their contribution in experimental EBA. Regarding CXCL1 and CXCL2, an increased expression was noted in the skin of mice with experimental EBA. In detail, CXCL1 mRNA expression was 50-fold increased comparing lesional to nonlesional skin samples from corresponding anatomical sites from mice with immunization-induced EBA. This was even more pronounced for CXCL2, where an increase over 1,500-fold was noted. Blockade of CXCL1 and CXCL2 functions by oral administration of allosteric CXCR1 and 2 inhibitors (DF2156A) dose dependently, but also strain-dependently, impaired the induction of skin blistering in mice induced by transfer of anti-COL7. In a more therapeutic setting, administration of DF2156A improved clinical EBA manifestation after disease onset in immunization-induced EBA. Compared to high doses of systemic corticosteroids, and inhibition of CXCR1 and 2 was equally effective [127]. Hence, CXCL1 and CXCL2 modulate blister formation in experimental EBA and thus are potential novel therapeutic targets for the treatment of EBA (see Section 14). 

Granulocyte macrophage colony-stimulating factor (GM-CSF) stimulates the production, induces migration, and activates many haematopoietic cells. GM-CSF is secreted as a glycosylated protein and consists of a single polypeptide chain. Its receptor consists of a specific GM-CSF binding subunit (CSF2R*α*), and a common signal-transduction subunit (CSF2R*β*) which is shared with other cytokines, such as IL-3 and IL-5 in humans [135, 136]. GM-CSF has diverse effects on different inflammatory diseases: application of recombinant GM-CSF improved Crohn's disease in patients [137, 138]. In experimental contact hypersensitivity, characterized by an increased GM-CSF expression [139], blockade of this cytokine had no effect on disease manifestation [140]. In contrast to these observations, blockade of GM-CSF in experimental models of arthritis [141–143], experimental autoimmune encephalitis [144], psoriasis [145], and nephritis [146] protected from disease induction. To test if the observed increased GM-CSF expression in experimental EBA is of functional relevance, anti-COL7 IgG was injected into GM-CSF-deficient mice or mice treated with a function-blocking GM-CSF antibody. Compared to appropriate controls, induction of experimental EBA was impaired if GM-CSF function was blocked. On the molecular level, GM-CSF was required to recruit neutrophils from the bone marrow into the blood and from blood into the skin. In addition, GM-CSF also preactivated the neutrophils, leading to an enhancement of immune complex induced neutrophil activation. Therapeutic blockade of GM-CSF in mice with already established immunization-induced EBA showed beneficial therapeutic effects [128].

IL-6 is predominantly known as a proinflammatory cytokine. This assumption is based on the observation of a correlation of IL-6 serum levels with disease activity in rheumatoid arthritis, Crohn's disease, asthma, and psoriasis [147–150]. Experimentally, the inhibition of IL-6 suppresses the development of inflammatory disease in vivo [151–154] thus highlighting the proinflammatory role of this cytokine. The relevance of IL-6 in contributing to the pathogenesis of inflammatory disease is further supported by the therapeutic effect of tocilizumab on patients with rheumatoid arthritis [155]. Interestingly, most of these proinflammatory effects of IL-6 are mediated by IL-6 transsignaling [156] as (i) protection from experimental arthritis in IL-6-deficient mice could be only restored by the injection of a fusion protein of IL-6 and the sIL-6R (hyper-IL-6), but not by IL-6 alone [157], and (ii) the treatment of experimental arthritis in wild type mice with a fusion protein consisting of the entire extracellular portion of gp130 fused to the Fc region of human IgG1 132 (sgp130) leads to a similar (protected) phenotype as observed in IL-6-deficient mice [157–159]. Contrary, IL-6 may also exert profound anti-inflammatory properties in some experimental models [160–163]. Due to these opposing effects of IL-6 in different inflammatory conditions, the functional relevance of the elevated IL-6 concentrations in experimental EBA was evaluated. In both IL-6-deficient mice and wild type mice treated with anti-IL-6, clinical EBA severity was significantly increased compared to the respective controls. On the contrary, administration of recombinant IL-6 dose-dependently impaired the induction of experimental EBA by the transfer of anti-COL7 IgG. These effects were at least partially due to an induction of IL-1ra by IL-6 and exclusively mediated by classical IL-6 signaling [123]. Therefore, IL-6 has a strong anti-inflammatory role in autoantibody-induced tissue injury in EBA. Despite the fact that the effect of IL-6 on autoantibody production in EBA is not known at the moment, great caution should be taken when considering blocking IL-6 functions in patients with EBA (and possibly other related diseases such as bullous pemphigoid). 

Of the other cytokines with noted increased expression in experimental EBA [123, 124], no functional data has been obtained so far (Table 5). 

### 12.6. Complement Activation Is Required for Blister Formation in Experimental EBA

The complement system consists of circulating proteins, which upon activation initiate a highly controlled cascade that is an integral part of the innate humoral immune response [164]. Uncontrolled activation of the complement system has been shown to contribute to the pathogenesis of several chronic inflammatory diseases, such as arthritis [165], asthma [166], and bullous pemphigoid [167]. In line with these observations, C5-deficient mice were completely protected from induction of experimental EBA by antibody transfer [44]. A more detailed investigation of the role of complement in EBA showed that the alternative complement activation is predominantly required for mediation of skin blistering in experimental EBA. Mice deficient for C1q, in which classical complement activation does not take place [168], showed a small and partial but significant protection from EBA induction. In contrast, mice lacking MBL expression, in which most of the lectin activation pathway does not occur [169], did show a similar EBA phenotype compared to wild type controls [129]. Collectively, this data points towards a significant contribution of the complement system in the pathogenesis of the effector phase, that is, autoantibody-induced tissue injury. However, data from asthma [166] suggested that during the initiation of immune responses, complement activation may have immunomodulatory effects. Therefore, the contribution of complement to the early phases of EBA pathogenesis has yet to be defined. 

### 12.7. Integrin-Dependent Extravasation of Leukocytes into the Skin

In order to appropriately locate the limited number of leukocytes to sites of inflammation, leukocytes constantly migrate from the blood to lymphoid organs, as well as to sites of inflammation. This process of leukocyte extravasation is tightly regulated and primarily mediated by adhesion molecules and cytokines. Leukocyte extravasation is initiated by interactions of endothelial adhesion molecules of the selectin family with their leukocytes counterparts. This interaction allows rolling and tethering of leukocytes along the endothelium. During this process, cytokines can lead to the activation of leukocyte integrins, which then mediate firm adhesion. Ultimately, leukocytes leave the vasculature through interactions mediated by junctional adhesion molecules [170–175]. In the effector phase of EBA, that is, autoantibody-induced tissue injury, neutrophils are the main effector cells, as their depletion completely protects from disease induction [126]. Regarding the contribution of adhesion molecules in the pathogenesis of EBA, only one study has been performed. In brief, CD18 deficient mice were completely resistant to autoantibody transfer-induced EBA [126]. However, based on the multistep process of leukocyte extravasation to the skin, it is tempting to speculate that other adhesion molecules also mediate neutrophil extravasation into the skin after the binding of COL7 autoantibodies. 

### 12.8. Fc Receptor Binding Initiates Autoantibody-Induced Tissue Injury in EBA

After their extravasation into the skin, neutrophils bind to the Fc fragments of the tissue-bound anti-COL7 antibodies. The importance of Fc/Fc receptor binding has been demonstrated both in vitro and in vivo: (i) only IgG antibodies but not corresponding F(ab)2 fragments (despite equal binding ability to the skin) induce dermal-epidermal separation in cryosections in the presence of neutrophils [60], (ii) in line, in mice, only anti-COL7 IgG but not the corresponding F(ab)2 fragments induces skin blistering [44, 64], (iii) anti-COL7 IgY, which does not bind to murine Fc receptors, fails to induce skin blistering when transferred into mice [104], and (iv) removal of terminal sugar residues on IgG, which alters their binding acidity to Fc gamma receptors [176] also completely protects mice from EBA induction by antibody transfer [113]. In mice, three different activating Fc gamma receptors (FcgRs) and one inhibitory FcgR have been described: FcgRI, FcgRIII, and FcgRIV are activating FcgR differing with their Fc-binding avidity. FcgRIIB is the only so far described inhibitory FcgR in mice [177]. Data from previously detailed experiments indicated that activating FcgR is required for blister formation in EBA. Furthermore, expression profiling from lesional EBA skin of mice showed an increased expression of FcgRIV [124]. Mice lacking the common, signal-transducing *γ*-chain of all activating FcgR were completely protected from EBA induction by antibody transfer. Further analysis, using different knock-out mice and FcgR-function-blocking antibodies, identified FcgRIV as the sole mediator of tissue injury in EBA. Blockade of FcgRI, FcgRIII, or even both receptors combined had no effect on EBA manifestation [124]. In humans, albeit the data has been obtained using sera from BP patients, FcgRIIA and FcgRIIIB mediate Fc binding of effector cells of immune complexes [73]. Furthermore, blockade of the FcgRIIB showed that this FcgR protects mice from EBA development [124]. This observation is in line with those obtained in animal models of arthritis and Goodpasture's syndrome [178, 179]. Interestingly, in experimental BP induced by the transfer of rabbit anti-mouse type XVII collagen into neonatal mice, the FcgRIIB had no effect on disease manifestation [180], which may be due to the young age of the mice. 

### 12.9. Complex Interaction of Fc Receptor Ligation and Complement Activation

**Figure 5:**
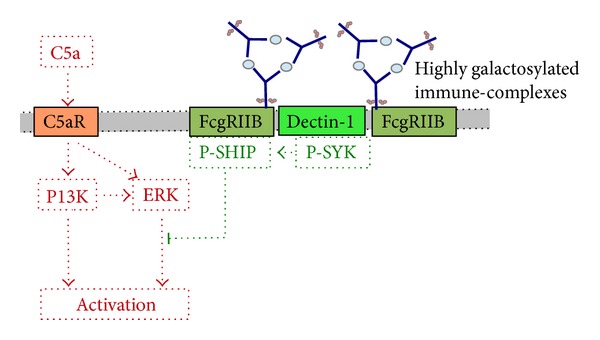
Modulation of complement-driven inflammation through FcgRIIB and dectin-1. Highly galactosylation IgG1 immune complexes bind to FcgRIIB. Galactosylation links FcgRIIB to dectin-1 resulting in tyrosine phosphorylation of the ITAM-like motif downstream of dectin-1 and transient phosphorylation of Syk. This pathway inhibits C5a-mediated ERK1/2 phosphorylation and several cellular effector functions of C5aR.

Interestingly, it had been noted that the complement system can affect the expression and function of FcgR [181, 182]. Hence, complement activation in EBA is thought to increase the threshold for the activation of FcgR expressing cells. It had so far been unclear if IgG-mediated signaling through FcgR is capable to modulate complement-mediated effector functions. Interestingly, injection of IgG1 immune complexes reduced C5a-mediated neutrophil migration into the peritoneum in mice. Detailed analysis of this observation showed galactosylated IgG1 immune complexes through binding to the inhibitory FcgRIIB and dectin-1 block C5aR-mediated proinflammatory events in neutrophils (Figure 5). Thus, the administration of galactosylated IgG1 immune complexes also blocked EBA manifestation in mice [130]. This observation highlights the complexity of autoantibody-induced tissue injury in EBA. 

### 12.10. Neutrophil Activation

After binding the activating FcgR to the immune complexes located in the skin, this must be signaled in order to provoke a biological response from neutrophils. In macrophages and neutrophils, the Syk protein tyrosine kinase (SYK) is essential for FcgR signaling [183]. Hence, several SYK inhibitors are currently developed and tested for the treatment of several chronic inflammatory diseases [184]. Downstream of SKY, the Akt, ERK1/2, and p38 MAPK phosphorylation have been demonstrated to be involved in the signaling pathways after SYK phosphorylation [185, 186]. In vitro, neutrophil activation can be blocked by inhibition of either Akt, ERK1/2, p38 MAPK phosphorylation. In detail, blockade of either of these pathways dose-dependently inhibited ROS release, as well as dermal-epidermal separation. In contrast, only inhibition of p38 MAPK phosphorylation led to reduced IC-induced degranulation of neutrophils [131]. In vivo, inhibition of ERK1/2 or p38 MAPK phosphorylation impairs induction of skin blistering in antibody transfer-induced EBA [131]. In addition, PI3K beta has recently been identified to be crucial for signal transduction in immune complex activated neutrophils. In detail, blockade of PI3K beta substantially inhibited the production of ROS from neutrophils, and in both antibody transfer-induced EBA and the K/BxN arthritis model, mice were protected from disease induction. In experimental EBA, the effect of PI3K beta could be linked to radiosensitive cells [61]. 

### 12.11. Mediators of Blister Formation

Neutrophil activation leads to several biological responses. The release of ROS and proteases is a prominent feature of neutrophil activation [187]. In experimental models of EBA, ROS production has been demonstrated to be crucial for blister formation. In vitro, neutrophils obtained from patients with chronic granulomatous disease, which are not capable to mount an oxidative burst [188], fail to induce dermal-epidermal separation when incubated on cryosections of human skin with deposits of anti-COL7 IgG at the basement membrane [126]. Furthermore, mice lacking cytosolic factor 1 are also completely protected from antibody transfer-induced EBA [126]. In addition to ROS, gelatinase B and elastase have been identified as crucial mediators of dermal-epidermal separation in EBA [132].

### 12.12. Flii Is a Key Regulator of Resolving Autoantibody-Induced Tissue Injury in EBA

The actin remodeling protein, Flightless I (Flii), has recently been shown to contribute to resolving skin blistering in experimental models of EBA. This work was prompted by previous observations in models of wound healing that demonstrated that reducing Flii expression leads to an improved wound healing. In contrast, Flii over expression resulted in impaired wound healing [189]. In autoantibody transfer-induced EBA, induction of EBA leads to an increased cutaneous Flii expression. This reduced Flii expression in Flii+/− mice significantly impaired blister formation in experimental EBA [133]. Subsequent studies demonstrated that topical treatment with Flii-neutralizing antibodies has therapeutic effects in this model of EBA [134]. These observations point towards significant contribution of pathways that are involved in resolving cutaneous involvement. Therefore, EBA may not only manifest when many proinflammatory stimuli are present, but also (maybe indeed only) when the balance of proinflammatory, anti-inflammatory, and resolving pathways are in a disbalance, which polarizes towards proinflammatory mechanisms. 

## 13. Diagnosis of EBA

The diagnosis of EBA is based on the clinical presentation, the detection of tissue-bound antibodies by direct IF microscopy, and the detection of circulating antibodies directed against COL7 and/or a u-serrated pattern in direct IF microscopy. Additional tests such as transmission electron microscopy or antigen mapping may be performed in unclear cases. 

### 13.1. Clinical Presentation

If the history and clinical presentation of the patient prompts EBA as a potential differential (see Section 1), subsequent laboratory analysis has to be performed to exclude or diagnose EBA. At a minimum, this includes obtaining a lesional biopsy for H&E staining and a perilesional biopsy for direct IF microscopy. In addition, serum analysis for the detection of autoantibody reactivity should be performed. In my personal opinion, other tests should be reserved for special indications. 

### 13.2. Detection of In Vivo Bound Antibodies

**Figure 6:**
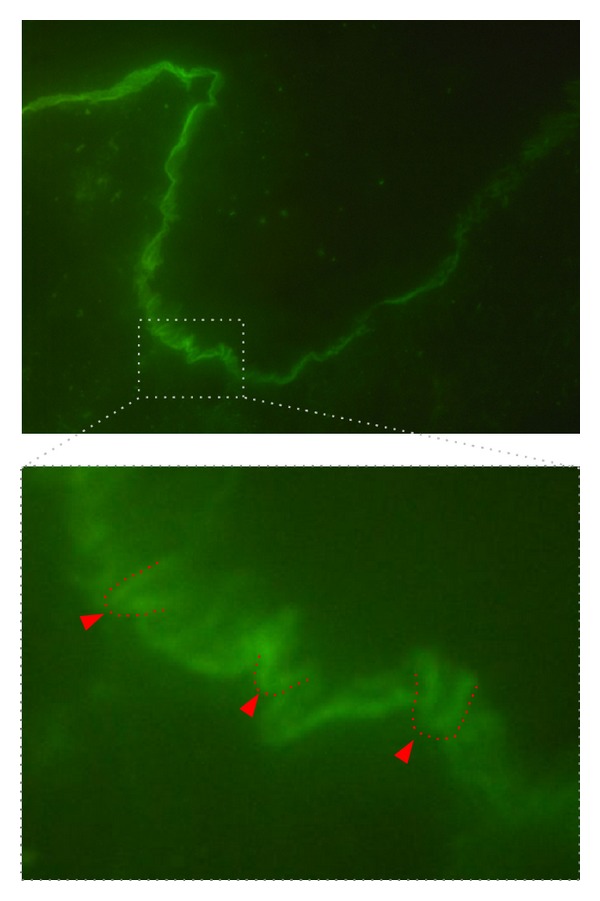
u-serrated pattern in direct IF microscopy in EBA. Direct IF microscopy from perilesional EBA skin (staining for IgG, 400x original magnification). A linear binding along the dermal-epidermal junction is evident. The insert further magnifies the u-serrated binding of the autoantibodies (highlighted in red), which can also be observed in the original direct IF microscopy photograph.

The detection of a linear IgG and/or IgA deposition along the basement membrane in a perilesional skin lesion from the patient is observed in almost all EBA cases. In detail, these deposits can be detected in at least 93% of all EBA patients. If the direct IF sections are observed at high magnification, n- and u-serrated patterns can be differentiated. The n-serrated pattern is seen in several subepidermal AIBD, but the u-serrated pattern is unique to EBA (Figure 6). If this criterion is included for EBA diagnosis, the diagnostic sensitivity can be greatly increased [6, 190–192]. 

### 13.3. Detection of Circulating Anti-COL7 Antibodies

Although it can be trained with a web-based program [191], pattern analysis in direct IF microscopy requires an experienced histopathologist. Therefore, detection of circulating anti-COL7 antibodies is a suitable alternative and/or provides additional information to base the diagnosis on. For the detection of anti-COL7 IgG antibodies, three different ELISA systems have been developed, and two of these are commercially available [193–195]. Autoantibodies directed against COL7 can also be specifically detected by IF microscopy using fixed, NC1-expressing HEK293 cells as substrate [195] or by western blotting with NC1 as substrate [56]. In addition, reactivity with a 290-kDa-sized protein of dermal extract and binding to the blister floor in salt-split skin in indirect IF microscopy point towards anti-COL7 antibodies. However, in the later cases, reactivity to laminin gamma-1 and laminin-332 has to be excluded [5]. 

### 13.4. Other Diagnostic Tests for the Diagnosis of EBA

#### 13.4.1. Histopathology

For histopathology, the biopsy has to be obtained from lesional skin. In most specimen, subepidermal blister formation is observed. The degree of the dermal leukocyte infiltration, as well as its composition shows great variability. However, to the author's knowledge, no detailed analysis of skin sections from EBA patients has been performed. In summary, the histology in EBA is not pathognomonic but adds to the overall clinical picture, and thus, a skin biopsy should be obtained if EBA is suspected. 

#### 13.4.2. Transmission Electron Microscopy

Routine electron microscopy shows blister formation situated in the dermis leaving the basal lamina in the roof of the blister [196]. Transmission electron microscopy is however only used in unclear cases and in specialized centers. 

#### 13.4.3. Immunoelectron Microscopy

Direct immunoelectron microscopic examination of skin of EBA patients shows the presence of immune deposits in the anchoring fibril zone, just beneath the lamina densa. This localization is distinct from the deposits in other AIBD [196–198]. Immunoelectron microscopy is considered the gold standard for EBA diagnosis. However, based on practicability and availability, other methods, for example, u-serrated pattern analysis and the combination of direct IF microscopy with the detection of circulating autoantibodies, are more frequently used [199, 200]. 

#### 13.4.4. Overlay Antigen Mapping

Another elaborate method for the diagnosis of EBA is overlay antigen mapping. Here, perilesional skin specimens from patients are stained for several components of the basement membrane zone. Based on the location of the tissue-deposited autoantibodies (in relation to the other stained structures), this allows to differentiate EBA from other AIBDs.

### 13.5. Recommended Additional Diagnostic Workup in Patients with EBA

Patients with EBA should be treated by a dermatologist in a multidisciplinary approach. The importance of involving other specialties is based on the findings that EBA is associated with inflammatory bowel disease [201, 202]. Hence, a gastroenterologist on a regular basis should consult EBA patients. Furthermore, as the study by Dr. Luke and colleagues documented a high incidence of (sub)clinical mucosal and eye involvement [25], the respective specialties should also be involved in the treatment and care of EBA patients. 

## 14. Treatment of EBA

Due to the low prevalence, no controlled clinical trials on the treatment of EBA have been performed. Current recommendations for EBA treatment are therefore solely based on the clinical expertise by clinicians specialized in AIBD [203, 204]. In addition, the clinical phenotype and the disease severity have to be taken into account when selecting treatment for EBA patients. However, the increased understanding of the diseases' pathogenesis has identified several potential therapeutic targets. The following paragraphs describe the advantages and disadvantages of the currently applied EBA treatments and discusses the possibility of targeting those of the identified targets, where drugs modulating the respective pathways are already available. However, and of note, the treatment of EBA is challenging. In a cohort of 30 EBA patients, who were initially treated with a combination of methylprednisolone, dapsone, and colchicine, remission was achieved after a median of 9 months on therapy. Long-term followup of these patients showed complete remission in 46% and incomplete remission in another 46% of the patients 6 years after initiation of treatment [7]. Overall, most experts recommend colchicine as first line treatment, as it has fewer adverse events than most of the other medications used for EBA treatment. Below, all drugs used to treat EBA are shortly introduced. The comment at the end indicates the author's personal opinion. 

### 14.1. Currently Applied Treatments for EBA

#### 14.1.1. Corticosteroids

Most EBA patients are treated with systemic corticosteroids. Initial doses range from 0.5 to 1.5 mg/kg per day [3]. In most cases, corticosteroid treatment is combined with other immunosuppressive/modulatory agents to lower the corticosteroid dose. These include almost all of the treatment modalities described below. As steroid treatment often leads to improvement of EBA, it has to be considered as effective. Furthermore, corticosteroid treatment is also effective in antibody transfer-induced EBA in mice [131]. However, due to its known high number of adverse events, other treatment modalities should be taken into consideration before systemic corticosteroid treatment whenever possible. Furthermore, it is tempting to speculate that patients with inflammatory-type EBA respond better to corticosteroids compared to patients with noninflammatory EBA.

#### 14.1.2. Methotrexate and Azathioprine

Both compounds are used as steroid-sparing agents. There are no reports on single use of these compounds in EBA patients. Furthermore, no data is available documenting the effectiveness of either methotrexate or azathioprine as steroid-sparing agents in EBA. Therefore, both compounds should be used only in the treatment of refractory cases. 

#### 14.1.3. Cyclosporine

Since its first use in 1987 for EBA [205], cyclosporine has been used in a total of 11 EBA patients [203, 206, 207]. In all patients, the use of cyclosporine was reported to have improved EBA. Of note, in at least 3 patients, cyclosporine was used as monotherapy, leading to remission of EBA. Hence, cyclosporine is most likely an effective treatment of EBA. 

#### 14.1.4. Colchicine

Colchicine has been used in well over 40 EBA patients [7, 17, 208, 208–212]. As most reports state beneficial effects of colchicine, it should be used as first line treatment for patients with (inflammatory-type) EBA. 

#### 14.1.5. High Dose Intravenous Immunoglobulin (IVIG)

Immunoglobulin G preparations, isolated from human serum, have been used as substitution treatment for patients with antibody deficiencies and severe infections for many decades [213]. Later, intravenous application of high doses of IgG (IVIG) has been established as an effective therapy for many autoimmune diseases, including immune thrombocytopenia (ITP), Guillain-Barre syndrome, multiple sclerosis, myasthenia gravis, pemphigus disease, and Kawasaki disease [214–218]. Until 2010, 12 patients with extensive treatment-resistant EBA had been treated with IVIG, and the reported response was usually favorable [219]. In 2012, results on additional 10 IVIG-treated EBA patients were reported. These patients received IVIG in addition to the previous medication that had failed to control the disease. These earlier drugs were withdrawn over a 5–9-month period, and IVIG was continued as monotherapy for a total duration of 30–52 months. In all 10 patients, a satisfactory clinical response was observed, and during followup (29–123 months), no relapse was observed [220]. Collectively, this data strongly supports the assumption that IVIG is an effective treatment option for EBA. 

#### 14.1.6. Dapsone

Several case reports have stated the use of dapsone in EBA [7]. Of note, dapsone monotherapy has been shown to be effective in one EBA patient [221]. Further data, like for all applied treatments in EBA, is needed before final conclusions of the efficacy of dapsone can be drawn.

#### 14.1.7. Cyclophosphamide

Cyclophosphamide has been rarely used in EBA. Therefore, no interpretation on its efficacy for controlling EBA can be drawn [222].

#### 14.1.8. Rituximab

Anti-CD20 treatment with rituximab has been increasingly used for the treatment of AIBD [91, 223, 224], and currently, a large phase III clinical trial compares rituximab treatment to general corticosteroid therapy treatment in patients with pemphigus (NCT00784589). Regarding EBA, 12 cases have been reported with rituximab treatment. In most cases, a favorable outcome was reported. Most patients treated with rituximab, several previous treatments had failed. In most cases, anti-CD20 was administered in an adjuvant setting. One patient, unresponsive to previous treatments, was treated with immunoadsorption to lower antibody titers rapidly, followed by rituximab monotherapy. This led to rapid and lasting clinical remission. Another patient was treated with additional rituximab as azathioprine monotherapy had not improved EBA. Approximately 2 weeks later, the patient died of bacterial pneumonia [225–235]. Hence, anti-CD20 treatment should be taken into account when selecting treatments for patients with either severe and/or relapsing EBA. 

#### 14.1.9. Plasmapheresis and Immunoadsorption

Although several reports and case report series have documented a beneficial effect of plasmapheresis and immunoadsorption in AIBD—especially in pemphigus [236], only very limited experience with this treatment modality has been described in EBA. Taken together, 3 cases of either plasmapheresis or immunoadsorption in EBA patients have been published so far, and in all cases, plasmapheresis and immunoadsorption have been used in addition to other treatments [228, 233, 237]. Despite the improvement that was reported in all 3 cases, the currently available data does not allow drawing a final conclusion on the effectiveness of these methods for EBA patients. 

#### 14.1.10. Extracorporeal Photochemotherapy

In principle, similar conclusions like for plasmapheresis and immunoadsorbtion can be drawn for extracorporeal photochemotherapy. Currently, data on 8 EBP patients successfully treated with extracorporeal photochemotherapy has been reported [16, 238–241]. Hence, this method seems promising for the management of treatment-refractory patients. 

### 14.2. Therapeutic Targets for Future EBA Therapy

Based on the increasing, but so far from complete, understanding of EBA pathogenesis, several potential therapeutic targets have been identified. This paragraph briefly describes those potential therapeutic targets, where compounds are already in use for other indications.

#### 14.2.1. Targeting the Generation of Antibodies

Several *HSP90 inhibitors* are in phase I-II clinical trials for oncology indications [242]. As the blockade of HSP90 prevented the onset of immunization-induced EBA and improved already established disease [84], blockade of HSP90 has also emerged as a potential therapeutic target for EBA. However, as HSP90 inhibition is associated with several adverse events, including treatment-related death [243], the dosing has to be adjusted in EBA patients, or less toxic HSP inhibitors such as TCLB-145 [84] must be used. To achieve this, detailed preclinical pharmacodynamics studies and dose-finding studies have to be performed. With the documented relevance of T cells for the generation of autoantibodies in experimental EBA [83] and the detection of COL7-specific T cells in (albeit few) EBA patients [92], T-cell depleting antibodies may be an option for severe and treatment refractory cases. Currently, several anti-CD3 and anti-CD4 antibodies are tested in inflammatory and malignant diseases [244–248]. 

#### 14.2.2. Removal of Autoantibodies

As an adjuvant treatment of AIBD (mainly pemphigus), *immunoadsorption* is performed [249]. Currently, a large phase III trial evaluates the efficacy and safety of immunoadsorption in pemphigus patients [250]. So far, experience with this technique is limited to few EBA patients (see Section 14), but overall immunoadsorption seems well suited to quickly reduce the levels of circulating autoantibodies, resulting in relatively rapid therapeutic response. Ultimately, as the autoantigen and the pathogenic relevance of autoantibodies are well documented, an antigen-specific immunoadsorption would be the ideal method to induce rapid clinical response in EBA patients. Antigen-specific removal of autoantibodies is well documented in dilative cardiomyopathy [251], and hence the principle seems applicable to other diseases, including EBA. 

#### 14.2.3. Modulation of Autoantibody-Induced Tissue Injury

The binding of *activating FcgR* to the immune complexes located in the skin is a key feature for autoantibody-mediated blister formation in EBA [124]. Hence, blockade of this interaction seems an attractive therapeutic approach. Currently, SM101, a soluble FcgRIIB which competes with FcgR expressed on immune cells for pathogenic immune complexes [252], is in phase II studies for primary immune thrombocytopenia (ITP) and systemic lupus erythematosus (SLE) [253]. In experimental models of lupus and arthritis SM101 prevented disease onset and/or improved already established disease [254, 255]. Hence, application of SM101 seems a promising treatment option for patients with treatment refractory EBA. As the lack of CD18 expression prevented neutrophil extravasation into the skin and subsequently clinical disease manifestation in experimental EBA [126], modulation of *leukocyte extravasation* could be targeted in EBA patients. Potential drug candidate is anti-alpha4 integrin (natalizumab), licensed for the treatment of multiple sclerosis [256]. Due to the observed progressive multifocal leukoencephalopathy under natalizumab treatment [257], risks and benefits have to be carefully considered, and patients must be monitored regularly. Other treatments targeting leukocyte extravasation, for example, anti-CD11a (efalizumab), have been withdrawn [258], have not been successful in sufficiently controlling skin inflammation [259, 260], or have not been tested in inflammatory skin conditions, such as KW-0761, which is used in the treatment of relapsed adult T-cell leukemia-lymphoma [261]. In principle, anti-CCR4 could be a therapeutic target in EBA, as CCR4 is involved in leukocyte recruitment into the skin [262]. An alternative to the targeted disruption of leukocyte extravasation by use of recombinant antibodies could be modified heparins. Besides its potent anti-coagulant activity, heparin has potent anti-inflammatory activities [263, 264]. Structural modifications of structurally defined glucan sulfates obtained by chemical modifications of neutral homoglycans produced by algae, bacteria, or fungi led to the production of compounds with reduced anticoagulant activity but stronger anti-inflammatory and/or antimetastatic activity compared to heparin [265]. One of these modified homoglycans, termed PS3, inhibits cutaneous inflammation [266].

In addition, selected cytokines such as *CXCL1* and *CXCL2* have been identified as novel therapeutic targets (see Section 7). Several compounds blocking CXCL1 and/or CXCL2 have been developed, for example, reparixin that enhanced pancreatic islet survival after transplantation in a phase 2 randomized, open-label pilot study [267]. In experimental models, a similar allosteric CXCR1/2 inhibitor prevented the onset of antibody-transfer-induced EBA and improved already established immunization-induced EBA [127]. Therefore, inhibition of CXCL1/2 by receptor-blockade seems a valid therapeutic approach for patients with treatment refractory EBA. *GM-CSF* also potently affects neutrophils functions. As detailed previously, blockade of this cytokine has beneficial effects both in antibody transfer- and immunization-induced EBA. GM-CSF has emerged as a target for rheumatoid arthritis, and three different anti-GM-CSF antibodies are currently evaluated in phase I-II clinical trials for this indication (MOR103, MorphoSys AG, NCT01023256; KB003, KaloBios Pharmaceuticals, NCT00995449; MT203, Takeda Pharma A/S, NCT01317797). Furthermore, the identification of the anti-inflammatory effects of IL-6, which is at least partially mediated by regulation of *IL-1ra,* makes use of recombinant IL-1ra (Anakinra). Anakinra is very effective in patients with Schnitzler's syndrome [268]. To the author's knowledge, Anakinra has not been used to treat AIBD or EBA patients. However, based on the efficacy of the compound in other inflammatory conditions and the preclinical data in experimental EBA, the use of compounds targeting IL-1 would be expected to have beneficial effects on EBA patients. 

## Figures and Tables

**Table 1:** Epidemiology and Ig reactivity in EBA.

Gender distribution	61% female 39% male

Age (median, 75 percentile)	47, 30–66 years*

Clinical phenotypes	Inflammatory phenotypes: 66% Classical (mechanobullous) phenotype: 33%

Ig reactivity (DIF positive)	IgG: 39% IgA: 19% IgG and IgA: 35% Not detected: 7%

*Black EBA patients with African descent have a significantly younger age when diagnosed compared to cohorts in Korea or the Netherlands. Abbreviations used: DIF: direct immunofluorescence microscopy. Data is based on 83 EBA patients [6–8].

**Table 2:** Demonstration of pathogenicity of antibodies directed towards type VII collagen.

Method	Protocol	Readout	Reference
Neutrophil activation in vitro	Incubation of isolated neutrophils with immune complexes of anti-COL7 antibodies and COL7	(i) ROS release (ii) Elastase release	[61]

Neutrophil activation ex vivo	Incubation of cryosections of human/mouse skin with antibodies against COL7 (serum, IgG, affinity-purified IgG from patients, monoclonal anti-COL7 antibodies, or rabbit anti-COL7 IgG)	(i) Deposition of formazan (ROS detection) (ii) Extent of dermal-epidermal separation	[60, 62]

Anti-COL7 IgG transfer into mice	(a) Repetitive injection of rabbit anti-mouse COL7 IgG into C57Bl/6, BALB/c, or BALB/c^nude^ mice	(i) Body surface area affected by skin lesions (ii) Several secondary endpoint measurements	[44]
	(b) Repetitive injection of rabbit anti-human COL7 IgG into SKH1 mice	Same as above	[63]
	(c) Repetitive injection of human anti-human COL7 IgG into SKH1 mice	Same as above	[64]
	(d) Repetitive injection of human affinity-purified (CMP) anti-human COL7 IgG into SKH1 mice	Same as above	[57]
	(e) Repetitive injection of human affinity-purified (Fn3-like domain) anti-human COL7 IgG into SKH1 mice	Same as above	[65]
	(f) Repetitive injection of rabbit anti-mouse COL7 IgG (vWFA2-like specific) into several in- or outbred mice	Same as above	[66]
	(g) Repetitive injection of rabbit anti-human COL7 IgG into COL7-humanized mice	Same as above	[67]

Immunization of mice with COL7	(a) Repetitive immunization of SJL/J, BALB/c, and Fc gamma receptor IIB-deficient mice (antigen located within the Fn3-like repeats)	(i) Autoantibody production (ii) Body surface area affected by skin lesions (iii) Several secondary endpoint measurements	[45]
	(b) Single immunization of SJL/J, B6.SJL-H2s, C57Bl/10.s, and MRK/MpJ mice (antigen located within the Fn3-like repeats)	Same as above	[68, 69]
	(c) Single immunization of SJL/J, B6.SJL-H2s, mice (antigen: vWFA2-like domain)	Same as above	[66]

Abbreviations used: COL7: type VII collagen, ROS: reactive oxygen species.

**Table 3:** Mechanisms leading to autoantibody production in EBA.

Mechanism	Demonstrated by	Reference
Genes within MHC locus	(i) HLA haplotype in EBA patients (ii) MHC association in experimental EBA	[8, 68, 80]

Genes outside MHC locus	(i) Overrepresentation of black-skinned patients in EBA (ii) Identification of quantitative trait loci (QTL) in experimental EBA	[8, 77]

CD4 T cells	(i) T-cell-deficient nude mice are resistant to induction of immunization-induced EBA (ii) Time-restricted depletion identifies CD4 T cells to be required for induction of immunization-induced EBA	[66, 83]

HSP90	Blockade of HSP90 prevents induction of and improves already established immunization-induced EBA	[84]

**Table 4:** Mechanisms leading to autoantibody-induced tissue injury in EBA.

Mechanism	Demonstrated by	Reference
Anti-COL7 IgG/A binding	Detection of anti-COL7 antibodies in patients, vast in vitro and in vivo (animal models) evidence	[125]
Genetic control	Diverse susceptibility of different inbred mouse lines	[66]
Neutrophils	Anti-Gr1 treatment completely protects from antibody transfer-induced EBA	[126]
Fc-fragment mediated effects	(i) Anti-COL7 IgG, but not corresponding F(ab)2 fragments, induces EBA in vitro and in vivo (ii) Anti-COL7 IgY fails to induce experimental EBA in mice (iii) Enzymatic removal of terminal sugar residues has preventive and therapeutic effects in experimental EBA in mice	[44, 60, 64, 104, 113]

Cytokines		
(a) CXCL1 and CXCL2	Blockade of the receptors has preventive and therapeutic effects in experimental EBA in mice	[127]
(b) GM-CSF	Genetic and pharmacological GM-CSF inhibition has preventive and therapeutic effects in experimental EBA in mice	[128]
(c) IL-6	IL-6 has anti-inflammatory effects in antibody transfer-induced EBA; IL-6 induced IL-1ra, which in turn counteracts proinflammatory events triggered by IL-1	[123]

Complement activation	C5-deficient mice are completely protected from antibody transfer-induced EBA; partial protection is observed in C1q-, Factor B-, or C5aR-deficient mice	[44, 129, 130]

Leukocyte extravasation	CD18-deficient mice are completely protected from antibody transfer-induced EBA	[126]

Fc gamma RIIB	Fc gamma RIIB-deficient mice show a significantly increased cutaneous blistering in antibody transfer-induced EBA	[124]

Fc gamma RIV	Fc gamma RIV-deficient mice are completely protected from antibody transfer-induced EBA	[124]

Neutrophil activation		
(a) PI3K beta	PI3K beta-deficient mice are partially protected from antibody transfer-induced EBA	[61]
(b) AKT	AKT impairs ROS released from immune omplex activated neutrophils	[131]
(c) p38	Pharmacologic inhibition of p38 phosphorylation partially protected from antibody transfer-induced EBA	[131]
(d) ERK1/2	Pharmacologic inhibition of ERK1/2 phosphorylation partially protected from antibody transfer-induced EBA	[131]

ROS	Neutrophil cytosolic factor 1-deficient mice are completely protected from antibody transfer-induced EBA	[126]

Elastase/Gelatinase B	Blockade of elastase or gelatinase B completely blocks dermal-epidermal separation	[132]

Flii	Overexpression of Flightless I (Flii) increases dermal-epidermal blistering in antibody transfer-induced EBA, and blockade of Flii improves blistering	[133, 134]

**Table 5:** Cytokine expression in experimental EBA.

Cytokine	Serum concentration	Cutaneous expression	Functional relevance	Reference
TNF-*α*	↑	↑	Proinflammatory (very moderate)	Unpublished
MIP-1*α*	↑	↑	none	Unpublished
GM-CSF	↑	↑	Proinflammatory (strong)	[128]
G-CSF	—	—	nd	
IL-1*α*	↑	↑	Proinflammatory	[123]
IL-1*β*	↑	↑	Proinflammatory	[123]
IL-2	Below detection limit	—	nd	
IL-3	—	—	nd	
IL-4	↑	nd	nd	
IL-5	—	nd	nd	
IL-6	↑	↑	Anti-inflammatory	[123]
IL-9	—	nd	nd	
IL-10	↑	nd	nd	
IL-12	—	nd	nd	
IL-13	—	nd	nd	
IL-17	↑	nd	nd	
IL-24	nd	↑	nd	
Eotaxin	—	nd	nd	
RANTES	↑	nd	nd	
IFN-*γ*	—	nd	nd	
MCP-1	—	nd	nd	
CXCL1 and CXCL2	↑	↑	Proinflammatory	[127]
CXCL3	nd	↑	nd	
CXCL5	nd	↑	nd	

Expression data is obtained from Samavedam et al. and from Kasperkiewicz et al. [123, 124]. Functional relevance was evaluated in the indicated publications. “↑”; “—” indicates statistically not significant difference in expression; “nd”: not done.
